# Description of Postoperative Void Patterns After Scheduled Cesarean Delivery Without Use of Routine Intraoperative Urinary Catheterization in a Large-Volume Safety Net Hospital

**DOI:** 10.7759/cureus.89260

**Published:** 2025-08-02

**Authors:** Kori A Baker, Rachel Anderson, Ellen Moore, Carolyn Valdez, Meredith Alston, Stefka Fabbri

**Affiliations:** 1 Obstetrics and Gynecology, University of Colorado School of Medicine, Aurora, USA; 2 Patient Safety and Quality, Denver Health, Denver, USA; 3 Obstetrics and Gynecology, Intermountain Health Saint Joseph Hospital, Denver, USA; 4 Obstetrics and Gynecology, Denver Health, Denver, USA

**Keywords:** cesarean delivery, intraoperative urinary catheter, postoperative recovery, postpartum urinary retention, urinary catheter, urinary retention, voiding

## Abstract

Background and objectives: While urinary catheters are widely utilized during cesarean delivery, little evidence exists to support the practice, and it may be associated with increased risk of urinary tract infections and unnecessary intervention. In this study we aim to describe postoperative voiding patterns and assess the prevalence of complications in patients undergoing scheduled cesarean delivery without an indwelling intraoperative urinary catheter.

Materials and methods: This is a prospective observational cohort of patients undergoing scheduled cesarean delivery at an urban safety-net teaching institution from April 2022 to April 2023. During the study period an institutional protocol was in place for non-use of routine urinary catheters at time of cesarean unless deemed necessary by the attending surgeon. Patients who underwent cesarean delivery in labor, had twin gestation, or needed a catheter postpartum, such as for administration of magnesium sulfate or for hemodynamic instability, were excluded. The primary outcomes included postoperative voiding patterns, including time to and volume of first spontaneous void, need for intervention for presumed urinary retention, and frequency of postoperative urinary retention. Secondary outcomes included postpartum hemorrhage, postpartum length of stay, postpartum pain scores, ureteral or bladder injury, urinary tract infection and unscheduled postpartum visits. Demographics, cesarean characteristics, voiding patterns, and complication rates were abstracted from the electronic medical record. Descriptive statistics were used to analyze the data.

Results: During the study period there were 3587 deliveries. Of those, 910 were cesarean deliveries, and 264 (29%) were non-laboring cesarean deliveries. A total of 215 (81.4%) of the cesarean deliveries were performed without a urinary catheter. Of those, the majority (73%) were scheduled elective repeats. After excluding postoperative catheter placement (N=16, 7.4%), the final cohort included 199 patients. The majority of patients were Latina (N=141, 70.9%), publicly insured (N=169, 84.9%), and at term (38.9±9 weeks). The most frequent indication for cesarean delivery was elective repeat (N=147, 73.9%), and the majority were performed under spinal anesthesia (N=193, 97.0%). The mean time to first postoperative void was 8.53 hours (SD 3.19) with median volume of 300 mL (IQR 175 - 400 mL). Thirty-nine (19.6%) patients had transient postoperative urinary retention based on inability to void by 10 hours (per institutional protocol) and catheterization volume ≥ 300mL. Only one (0.3%) patient required an indwelling catheter while hospitalized, and none were discharged home with a catheter. There were no intraoperative bladder or ureteral injuries. Postpartum length of stay, unscheduled outpatient visits, and postoperative urinary tract infection rates were comparable to those who had an intraoperative urinary catheter.

Conclusions: The mean time to the first postoperative void was 8.5 hours, which is longer than current algorithms for the management of postoperative urinary retention. Non-use of routine urinary catheter at the time of scheduled cesarean delivery was associated with transient urinary retention in 19.6% of patients; however, there was no increase in sequelae at time of discharge.

## Introduction

Indwelling urinary catheterization remains the standard of care for scheduled cesarean deliveries despite minimal supporting evidence for its routine use. Several studies have demonstrated benefits of limiting urinary catheter use in patients undergoing cesarean section either to complete avoidance of use or with immediate removal of the catheter postoperatively. When comparing cesarean delivery with limited urinary catheterization to routine catheter use, limited use was associated with two to 11 times lower risk of postoperative UTI [1-3]. In addition, several studies, including three randomized control trials, have demonstrated the benefits of non-use or immediate removal of urinary catheters including decreased time to ambulation and shorter hospital stays [1-8] without increase in intraoperative difficulty, bladder injury, or operative time [1,2,9,10]. With rates of cesarean delivery reaching 32.1% in the United States in 2021, the benefits of limiting catheter use should be explored [11].

One of the indications for intraoperative urinary catheterization is concern for postoperative postpartum urinary retention (PUR). The prevalence of PUR ranges from 3 to 7% of cesarean deliveries with rates varying widely due to inconsistent diagnostic criteria [12-14]. Studies have shown no difference in the rate of urinary retention when comparing use of catheterization for 12 hours postoperatively, non-use, or immediate postoperative removal of catheter [3,8-10]. Lastly, little data exist describing natural voiding patterns after cesarean delivery with non-use of urinary catheter.

In this prospective cohort our aim is twofold: 1) to describe the voiding patterns in patients undergoing scheduled cesarean delivery without indwelling urinary catheter; and 2) to evaluate the prevalence of perioperative complication when urinary catheters are not used at time of scheduled cesarean deliveries including urinary retention, need for catheterization, intraoperative bladder injury, postpartum hemorrhage, urinary tract infection and unscheduled urgent/emergent visits for bladder-related complaints. We hypothesize that most patients after scheduled cesarean delivery with no indwelling urinary catheter will void spontaneously by 10 hours postoperatively, and that the prevalence of perioperative complications will be similar to that of catheterized patients.

## Materials and methods

In this prospective observational cohort study, conducted at an urban safety-net teaching hospital, we evaluated patients undergoing scheduled cesarean delivery between April 2022 and April 2023. We excluded patients undergoing cesarean delivery in labor, twin gestations, those undergoing cesarean delivery at time of trauma and/or non-obstetric emergency, patients with known urinary retention, bladder dysfunction, or conditions predisposing to urinary retention such as spinal cord injury, as well as patients who had an immediate postoperative catheter placement for the administration of magnesium sulfate or for monitoring of hemodynamic status. 

In June 2021, the Department of Obstetrics and Gynecology at our institution adopted non-use of routine indwelling urinary catheters at time of cesarean delivery. For patients undergoing a scheduled cesarean delivery, the patient is encouraged to void within 30 minutes prior to the procedure and is monitored postoperatively for first void and signs of urinary retention. As this was a new practice, the decision to place intraoperative catheter was left at the discretion of the attending surgeon. Per the institutional guideline, patients are assessed for first void at eight hours postoperatively. If the patient had not voided and remained asymptomatic, ambulation and attempt to void were encouraged. If the patient had not voided by 10 hours postoperatively, the bedside registered nurse (RN) performed a bladder scan and notified the provider. Management at this juncture was individualized. 

The primary outcome of the study was to describe postoperative voiding patterns including the time to and volume of first void, the frequency of postoperative evaluation and/or intervention prior to voiding, and postoperative urinary retention. Time to first void was defined as the interval between completion of surgery in the operating room and first void. Time to intervention was defined as the interval between completion of surgery and time of first intervention, including bladder scan and/or straight catheterization. Urinary retention was defined as inability to void within 10 hours postoperatively and/or catheterization volume ≥ 300 mL based on institutional guideline and generally accepted volume definition for postoperative retention. Secondary outcomes included intraoperative bladder or ureteral injury, postpartum hemorrhage (quantitative blood loss ≥ 1 L within 24 hours of delivery), postpartum length of stay, postpartum pain score at 24 hours, postpartum unscheduled healthcare visits within six weeks postpartum, and postpartum urinary tract infection within six weeks. 

Data were abstracted from the electronic health record (EHR). Missing data prompted manual chart review. We used descriptive statistics to report demographics, cesarean delivery characteristics, postoperative voiding patterns and complications. We report the frequency of the primary and secondary outcomes. Student’s t-test was used for normally distributed data and χ2 for continuous and categorical variables with the level of significance set at 0.05. For non-normally distributed data, nonparametric tests are used to make comparisons. The study was reviewed and deemed exempt by the Colorado Multiple Institutional Review Board (COMIRB Exemption Protocol #: 22-0950).

## Results

**Figure 1:**
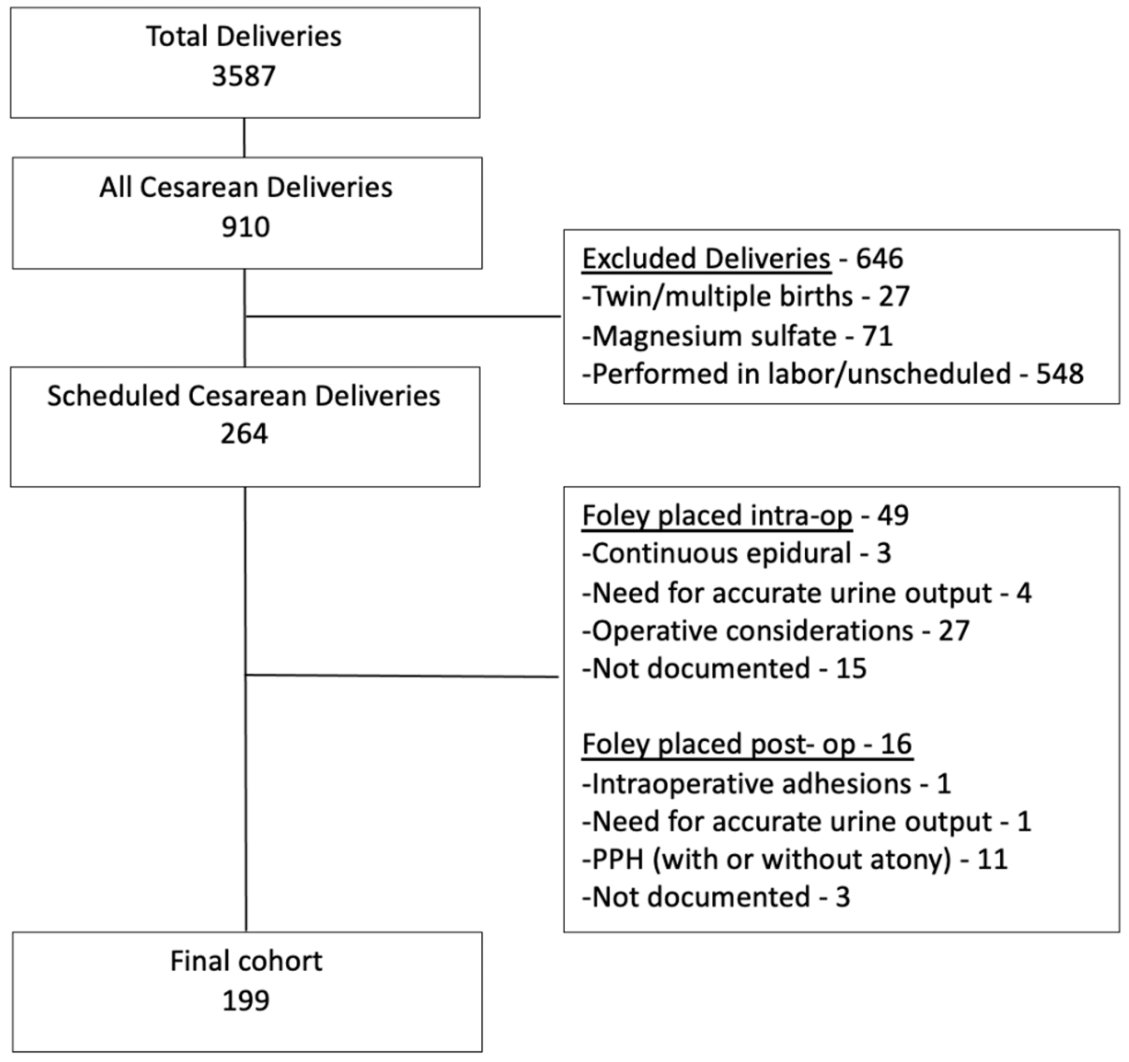
Study Population PPH: Postpartum hemorrhage

During the study period there were a total of 3587 deliveries of which 910 were cesareans. Of those, 264 (29.0%) were scheduled cesarean deliveries who met inclusion criteria and 215 (81.4%) were performed without urinary catheter. In 49 (18.6%) of the cases, an intraoperative catheter was used with the most frequently cited reason being operative considerations (N=27, 55%). In 16 cases (6.1%) a catheter was placed immediately postoperatively where the most common indication was postpartum hemorrhage (N=11, 68%). Thus, the final cohort included 199 patients (Figure 1). 

Demographic, obstetric, and cesarean characteristics are summarized in Table 1 and Table 2. The majority of the patients were Latina (N=141, 70.9%), publicly insured (N=169, 84.9%), and at term (38.9±0.9 weeks gestation). The most frequent indication for the cesarean was elective repeat (N=147, 73.9%), and the majority were performed under spinal anesthesia (N=193, 97.0%). In a sensitivity analysis comparing patients who underwent scheduled cesarean delivery with and without urinary catheter, those with intraoperative catheter were older (31.1 v. 33.0, p=0.0371), with higher body mass index (BMI, 33.2 v. 29.6 kg/m2, p=0.0012), with higher parity (3 v. 1, p<0.0001), and more likely to be Black or African American (32.7% v 13.5, p=0.0109). Further, in patients with intraoperative urinary catheter, the operative time (63.5 v. 47.3 minutes, p=0.0013) and intraoperative blood loss (896.1 v. 643.6 mL, p=0.0277) were significantly higher, and more patients had their cesarean performed under epidural anesthesia (12.2% v 1.9%, p<0.0001). 

**Table 1:** Demographic, obstetric, and operative characteristics of patients who underwent scheduled cesarean delivery without intraoperative urinary catheter ^1^Includes active genital herpes simplex virus (HSV), eclampsia/preeclampsia, history of 3c perineal laceration, shoulder dystocia, or traumatic birth, intolerance of sterile vaginal exam (SVE), vaginismus, maternal decompensation, placental abruption ^2^Includes category 3 fetal heart rate (FHR), cord prolapse, fetal Intolerance of labor, persistent category 2 FHR, terminal bradycardia ^3^Includes placenta previa and vasa previa

	Total Patients (N=199)
	Mean (SD)
Maternal age (years)	31.0 (5.9)
BMI (kg/m^2^) at first prenatal visit	29.7 (6.6)
Gestational age at delivery (weeks)	38.9 (0.9)
	Median (IQR)
Gravity	3 (2, 4)
Parity	1 (1, 2)
Race	N (%)
White or Caucasian	114 (57.3)
Black or African American	25 (12.6)
Asian	2 (1.0)
Other	57 (28.6)
Decline to answer	1 (0.5)
Ethnicity	N (%)
Hispanic, Latinx, or Spanish Origin	141 (70.9)
Not Hispanic, Latinx, or Spanish Origin	58 (29.1)
Health insurance coverage	N (%)
Public	169 (84.9)
Private	27 (13.6)
Uninsured	3 (1.5)
	Mean (SD)
Operative time (minutes)	46.9 (16.6)
Intraoperative quantitative blood loss (mL)	572.4 (280.1)
Primary indication for cesarean	N (%)
Breech	33 (16.6)
Elective repeat	147 (73.9)
Fetal macrosomia	1 (0.5)
History of uterine surgery	5 (2.5)
Malpresentation	2 (1.0)
Maternal indication^1^	2 (1.0)
Multiple gestation	3 (1.5)
Non-reassuring fetal status^2^	1 (0.5)
Placental indication^3^	5 (2.5)
Anesthesia type	N (%)
Spinal	193 (97.0)
Epidural	4 (2.0)
Combined Spinal and Epidural	2 (1.0)

**Table 2:** Demographic, obstetric, and operative characteristics of patients who underwent scheduled cesarean delivery with and without intraoperative urinary catheter ^1^Includes 16 patients who had urinary catheter placed immediately postoperatively. ^2^Includes active genital herpes simplex virus (HSV), eclampsia/preeclampsia, history of 3c perineal laceration, shoulder dystocia, or traumatic birth, intolerance of sterile vaginal exam (SVE), vaginismus, maternal decompensation, placental abruption ^3^Includes category 3 fetal heart rate (FHR), cord prolapse, fetal Intolerance of labor, persistent category 2 FHR, terminal bradycardia ^4^Includes placenta previa and vasa previa

	No Catheter (N=215)^1^	Catheter (N=49)	p
	Mean (SD)		
Maternal age (years)	31.1 (6.1)	33.0 (5.5)	0.0371
BMI (kg/m^2^) at first prenatal visit	29.6 (6.9)	33.2 (6.2)	0.0012
	Median (IQR)		
Gravity	3 (2, 4)	4 (3, 5)	0.0002
Parity	1 (1, 2)	3 (2, 3)	<0.0001
Race	N (%)		0.0109
White or Caucasian	121 (56.3)	24 (49.0)	
Black or African American	29 (13.5)	16 (32.7)	
Asian	4 (1.9)	2 (4.1)	
Other	59 (27.4)	7 (14.3)	
Decline to answer	2 (0.9)	0 (0.0)	
Ethnicity	N (%)		0.0001
Hispanic, Latinx, or Spanish Origin	150 (69.8)	20 (40.8)	
Not Hispanic, Latinx, or Spanish Origin	65 (30.2)	28 (57.1)	
	Mean (SD)		
Operative time (minutes)	47.3 (16.8)	63.5 (32.4)	0.0013
Intraoperative quantitative blood loss (mL)	643.6 (465.5)	896.1 (750.0)	0.0277
Primary indication for cesarean	N (%)		0.1732
Breech	35 (16.3)	1 (2.0)	
Elective repeat	157 (73.0)	41 (83.7)	
Fetal macrosomia	1 (0.5)	0 (0.0)	
History of uterine surgery	6 (2.8)	1 (2.0)	
Malpresentation	2 (0.9)	0 (0.0)	
Maternal indication^2^	4 (1.9)	1 (2.0)	
Multiple gestation	4 (1.9)	1 (2.0)	
Non-reassuring fetal status^3^	1 (0.4)	0 (0.0)	
Placental indication^4^	5 (2.3)	4 (8.2)	
Anesthesia type	N (%)		<0.0001
Spinal	208 (96.7)	38 (97.0)	
Epidural	4 (1.9)	6 (12.2)	
Combined Spinal and Epidural	2 (0.9)	4 (8.2)	
General	1 (0.5)	1 (2.0)	

**Figure 2:**
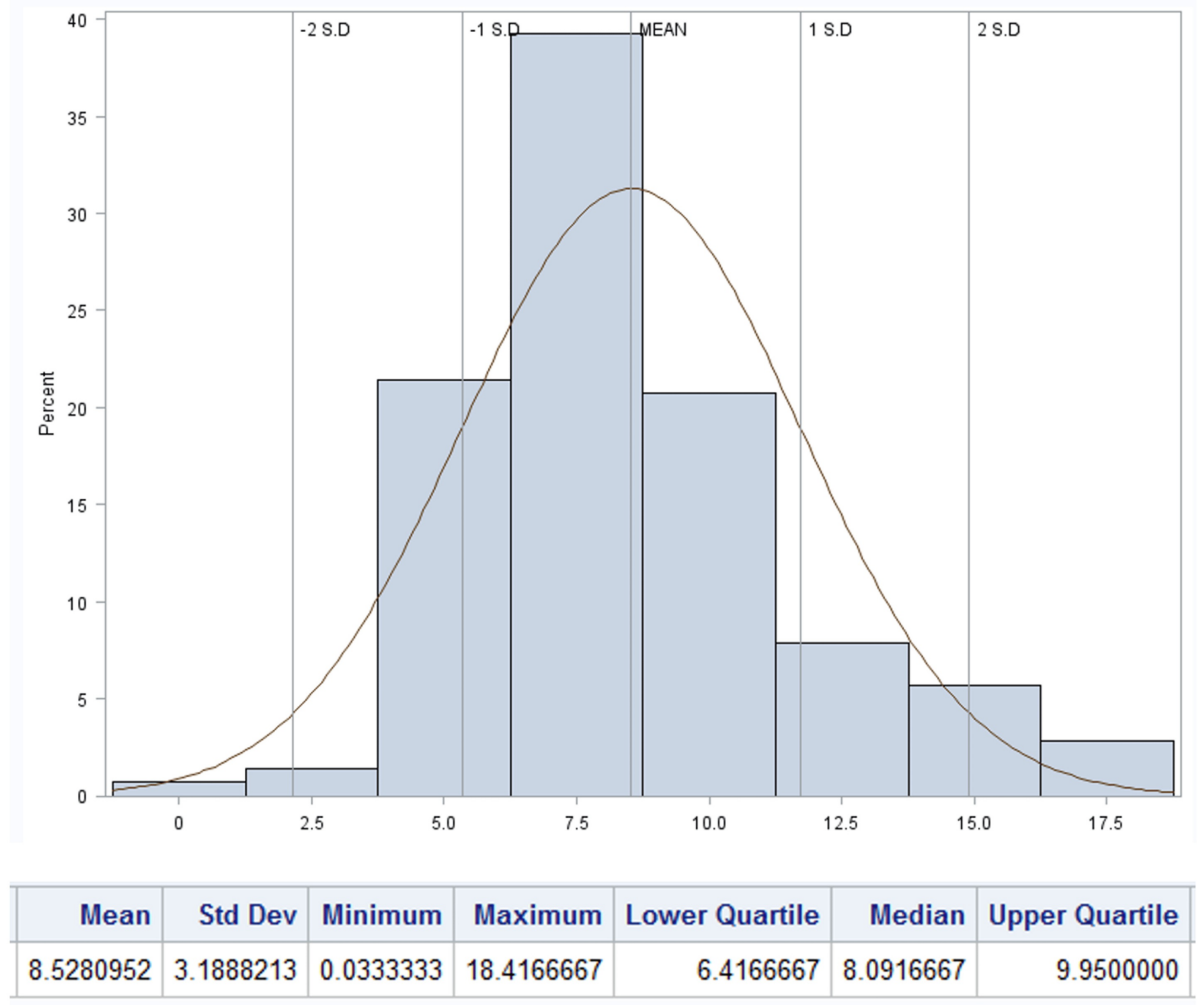
Distribution of time to first spontaneous postoperative void in patients undergoing scheduled cesarean without urinary catheter

**Figure 3:**
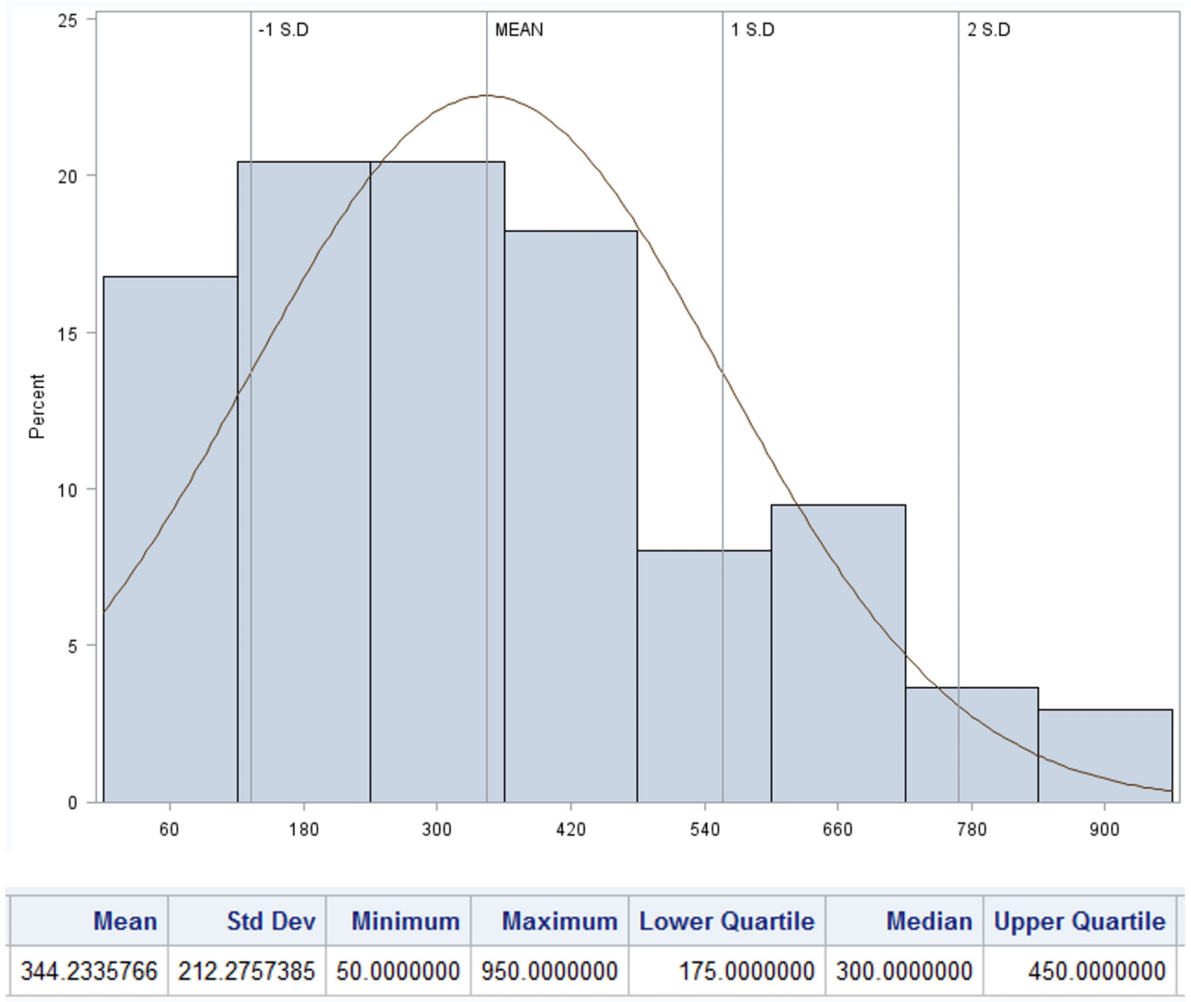
Distribution of volume of first spontaneous postoperative void in patients undergoing scheduled cesarean without urinary catheter

**Figure 4:**
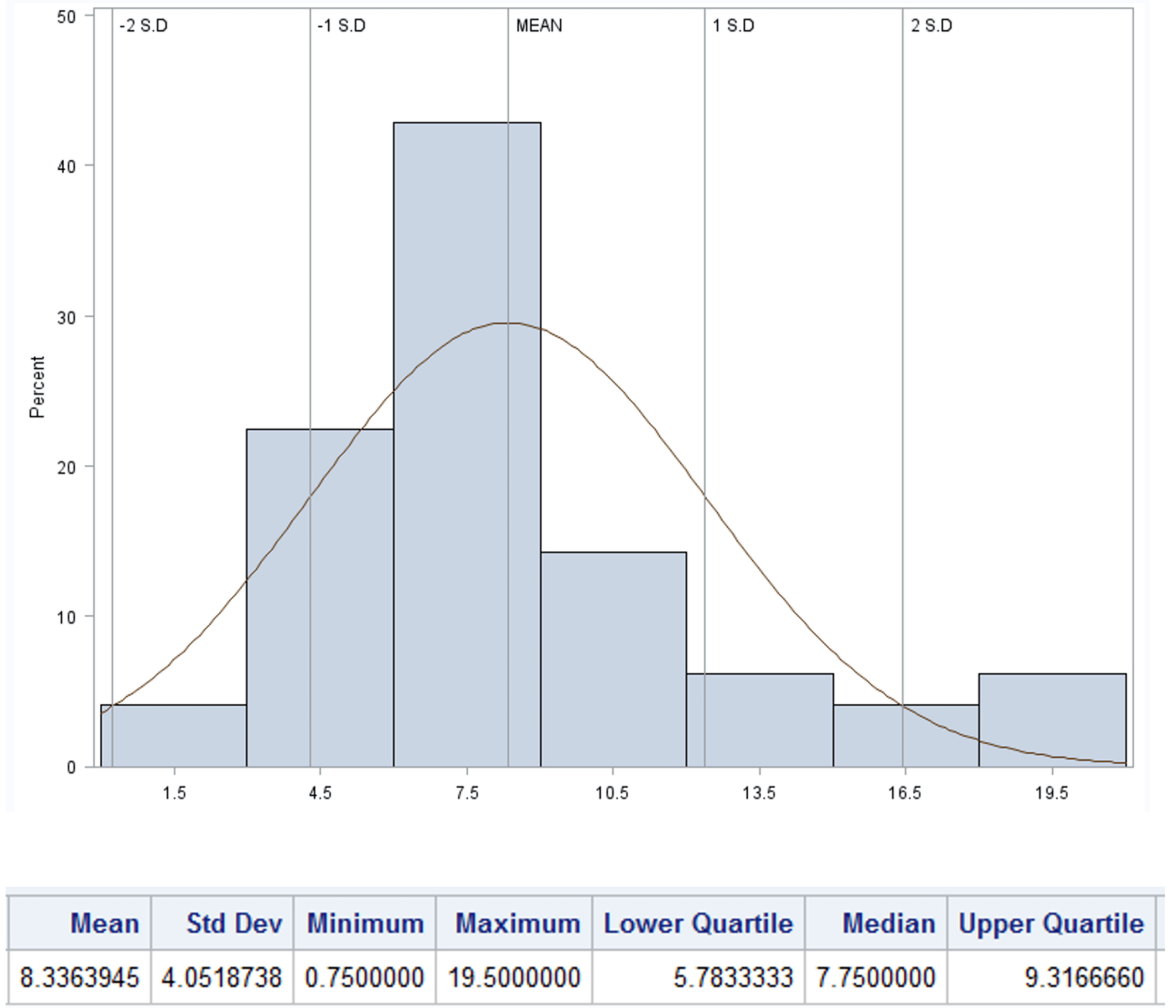
Distribution of time (in hours) to first spontaneous postoperative void in patients who underwent postoperative straight catheterization (N=55)

**Figure 5:**
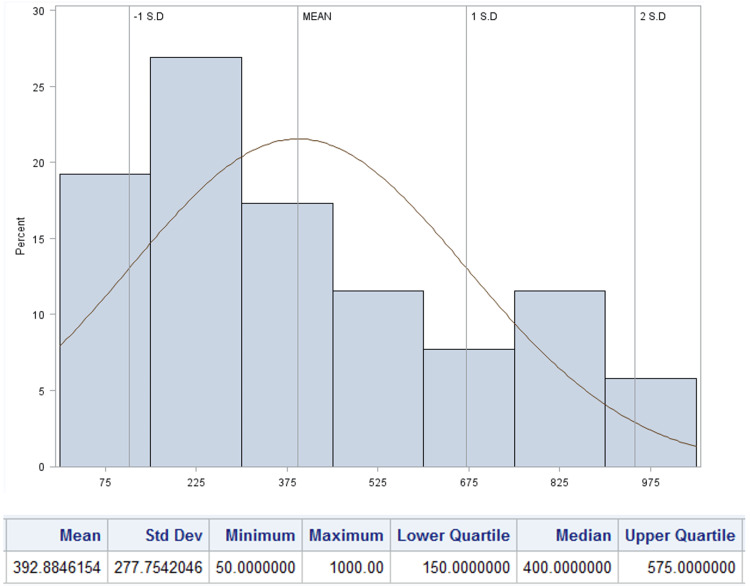
Distribution of volume (in mL) of first spontaneous postoperative void in patients who underwent postoperative straight catheterization (N=55)

Findings on postoperative voiding patterns, the need for provider evaluation, and the frequency of urinary retention are summarized in Table 3. The mean time to first spontaneous postoperative void was 8.53 hours (SD 3.19), and the median volume of the initial void was 300 mL (IQR 175 - 400 mL, Figures 2, 3, respectively). Seventy-six patients (38.2%) were evaluated by a provider in the postoperative period with the most frequent reason being inability to void (N=65, 85.5%). Notably, the mean time to evaluation was shorter, 6.0 hours (SD 4.3) than the institutional protocol at the time at 8.0 hours. Of the patients who underwent provider evaluation, 55 (27.6%) underwent straight catheterization with mean volume of 560.8 mL (SD 313.30). Two (1.0%) patients needed repeat catheterization. In patients who were straight catheterized the median time to their first spontaneous void was 7.8 hours (IQR 5.78 - 7.75) with median volume of 400 mL (IQR 150 - 575, Figures 4, 5). A total of 39 (19.6%) patients were diagnosed with urinary retention as previously defined. Of those, only one patient had insertion of indwelling urinary catheter, and none were discharged with urinary catheter.

**Table 3:** Description of voiding patterns in patients undergoing scheduled cesarean delivery without intraoperative urinary catheter ^1^Other – includes bleeding, incomplete emptying, and no urge to void

	Total Patients (N=199)
	Mean (SD)
Time to first post-op void, hrs	8.5 (3.19)
Volume of first post-op void, mL (median, IQR)	300 (175 – 400)
Time to intervention for inability to void, hrs	6.0 (4.3)
Need for provider evaluation, N (%)	76 (38.2)
Reason for provider evaluation, N (%)	
Inability to void	65 (85.5)
Other^1^	4 (5.3)
Not Documented	7 (9.2)
Bladder scan performed, (N, %)	85 (42.7)
Volume of bladder scan	
Initial, mL (N=85)	302.4 (208.7)
Repeat, mL (N=20)	435.3 (240.2)
Straight catheterization performed, (%)	55 (27.6)
Catheterization volume	
Initial, mL	560.8 (313.3)
Repeat, mL (N=2)	800 (0.0)
Time to straight catheterization, hrs	11.1 (2.6)
Time to void from straight cath, hrs (median, IQR)	7.8 (5.8 – 7.8)
Urinary retention, (N, %)	39 (19.6)

Secondary outcomes are summarized in Table 4. Patients who had intraoperative urinary catheter placed at the attending surgeon’s discretion were more likely to experience postpartum hemorrhage (35.4% v. 5.0%, p<0.0001) and had higher postoperative pain scores at 24 hours (5 vs 4, p=0.0344). There were no intraoperative bladder or ureteral injuries. There were no significant differences in postpartum length of stay, unscheduled healthcare visits after discharge, or urinary tract infections within six weeks postpartum.

**Table 4:** Secondary perioperative outcomes in patients who underwent scheduled cesarean delivery with and without intraoperative urinary catheter *Postpartum hemorrhage defined as blood loss >/= 1L

	No catheter (N=199)	Catheter (N=65)	p
	N (%)	N (%)	
Postpartum hemorrhage*	10 (5.0)	23 (35.4)	< 0.0001
Postpartum hemorrhage* due to atony	3 (1.5)	17 (26.2)	< 0.0001
	Median (IQR)	Median (IQR)	
Postpartum length of stay, days	2.2 (2.0, 2.3)	2.3 (2.1, 3.0)	0.0649
Postpartum pain score at 12 hours postoperatively	3 (1, 5)	4 (1, 6)	0.1240
Postpartum pain score at 24h postoperatively	4 (3, 6)	5 (3, 7)	0.0344
	N (%)	N (%)	
Postpartum unscheduled healthcare visits within 6 weeks postpartum (emergency department or urgent care)	44 (22.1)	22 (33.85)	0.0578
Postoperative urinary tract infection within 6 weeks postpartum	5 (2.5)	0 (0.0)	N/A

## Discussion

Principal findings

In this prospective cohort study of patients undergoing scheduled cesarean delivery without intraoperative urinary catheterization, the mean time to first spontaneous postoperative void was 8.53 hours (SD 3.19), and the median initial void volume was 300 mL (IQR 175-450 mL). Urinary retention, defined as inability to void by 10 hours postop or catheterization volume ≥ 300 mL, was observed in 19.6% of patients. Only 1.0% of patients required repeat catheterization, and none were discharged with an indwelling catheter. There were no bladder or ureteral injuries. Secondary outcomes, including postpartum length of stay, unscheduled postpartum healthcare visits, and UTI rates were similar between catheterized and uncatheterized patients, though the study was not powered to detect differences in these outcomes.

Results in the context of what is known

Limited data exist describing the natural voiding patterns in patients undergoing cesarean delivery without indwelling urinary catheter. Although the safety and necessity of urinary catheterization during cesarean delivery are increasingly debated, few U.S. studies have addressed this issue. Enhanced Recovery After Surgery (ERAS) guidelines recommend immediate catheter removal post-cesarean for patients not requiring strict urine output monitoring but do not provide clear guidance on intraoperative use or standardized perioperative protocols [15]. Our findings align with prior studies reporting mean times to first void between 4.64 and 8.76 hours [1,2,6,16]. Of note, while most studies recommend that patients void only upon feeling the urge, other protocols encouraged patients to void within a pre-specified time frame postoperatively, which may have hastened the timing of the first void [2]. 

First void volume in uncatheterized patients is less studied, with only one prior study reporting a mean of 180.51 mL [6]. Our study found a higher median volume of 300 mL, likely due to the longer time to first void compared to the shorter interval used by Acharya et al. 

Postoperative catheterization was performed in 27.6% of our patients without intraoperative catheter; however, the rate of urinary retention defined at our institution as inability to void by 10 hours postoperatively and/or catheterization volume ≥ 300 mL was 19.6% in the uncatheterized patient group. The higher catheterization rate when not meeting retention criteria is indicative of earlier, possibly unnecessary intervention and not following our newly implemented institutional protocol. Nonetheless, the urinary retention rate in our cohort is nearly double that of rates reported in other studies [6,15,17].

Rates of urinary retention in patients with intraoperative catheter placement is better studied, ranging from 0% to 24.1% [3,8,10,18-20]. Directly comparing these rates, however, is difficult due to widely varying definitions of postpartum urinary retention and the selection of different patient population where other studies have included higher rates of emergent cesarean, cesareans in labor and the use of general anesthesia. 

In our study, there were no bladder or ureteral injuries in uncatheterized patients, which may support the idea that a slightly filled bladder is better demarcated during the procedure and more easily avoided [1,2]. Studies by Acharya et al. and Senanayake similarly showed no cases of accidental cystotomy in their uncatheterized patient groups [1,6].

Finally, our rate of UTI was low, at 2.5% in patients who were not catheterized at time of cesarean, which is consistent with similar studies on non-catheterized patients [1,6,15,16]. Similar to urinary retention, variable definitions exist between studies to diagnose UTI, with some studies basing treatment on asymptomatic bacteriuria and others on culture-positive urine samples. When compared to their catheterized counterparts, patients who remained uncatheterized were less likely to develop UTI postpartum [17].

Clinical implications 

The data on post-cesarean section voiding patterns, especially in those performed without urinary catheter are limited. In addition, the variable definitions of postpartum urinary retention, many based on expert opinion, make the evaluation and standardization of clinical care challenging. Although studies exist to support the non-use of urinary catheterization during cesarean delivery, most focus on mean time to ambulation, duration of hospital stay, and incidence of UTI to support their conclusions [5-7,15,17]. Our study suggests that the mean time to void after scheduled cesarean delivery is considerably longer at 8.52 hours compared to adopted algorithms for the diagnosis and management of PUR between four and six hours [21,22].

Research implications 

Our study shows that the mean time to first spontaneous postoperative void was 8.53 hours (SD 3.19), thus questioning adopted definitions of PUR. Even though we did not observe a difference in intraoperative and postoperative complications, additional appropriately powered studies are needed. Further, future studies may consider cost analysis and possibly cost savings with restricted use of routine catheterization for scheduled cesarean deliveries.

Strengths and limitations 

The study has several limitations, including potential selection, ascertainment, and information biases. Selection and ascertainment bias stem from the fact that clinicians, unblinded to the intervention, determined the need for catheter placement. In a sensitivity analysis comparing patients who underwent scheduled cesarean delivery with and without urinary catheter, those with intraoperative catheter were older (p=0.0371) with higher body mass index (p=0.0012) and with higher parity (p<0.0001). Further, in patients with intraoperative urinary catheter, the operative time (p=0.0013) and intraoperative blood loss (p=0.0277) were significantly higher. The above findings indicated that providers were inclined to use intraoperative urinary catheter in cases anticipated to be more challenging based on patient obstetric characteristics, BMI, and history of prior cesareans. 

Information bias may also be present, as outcomes rely on accurate electronic medical record documentation and data capture. Patients with postoperative complications may have presented to an outside hospital or urgent care leading to underreporting of our secondary outcomes. Additionally, the lack of a concurrent control group limits our ability to fully account for confounders. Notably, the rate of postoperative catheterization (27.6%) was much higher than the catheterization performed for urinary retention criteria (19.6%). The mean time to intervention in our study was six hours, earlier than expected and inconsistent with institutional policy, which recommends nurse evaluation at eight hours postoperatively and provider evaluation by 10 hours. This deviation from protocol may have resulted in more frequent evaluations by nursing staff and, consequently, higher rates of intervention, some of which may have been unnecessary. The latter may be related to the adoption of this new practice where staff and providers felt uncomfortable allowing for the additional time past the accepted four to six hours postoperatively. Further, documentation of provider evaluation for urinary retention was inconsistent and unstandardized, possibly leading to underreporting.

Despite these limitations, the study has several strengths. It is one of the few studies to prospectively evaluate postoperative voiding patterns in patients undergoing scheduled cesarean delivery without the routine use of urinary catheter. Although additional studies are necessary, our findings challenge the accepted definitions of PUR based on inability to void at four to six hours. 

## Conclusions

This study demonstrates that non-use of indwelling urinary catheters during low-risk scheduled cesarean deliveries is a reasonable approach that does not increase the risk of adverse perioperative outcomes. While the majority of patients were able to void spontaneously without serious complications, 27.6% required single catheterization postoperatively to restore voiding function. These findings support a shift toward more conservative catheter use, particularly in selected low-risk patients, while emphasizing the importance of careful monitoring and timely intervention when needed. Furthermore, our results highlight the need to reevaluate current definitions of postoperative urinary retention, as existing criteria may not fully reflect the natural voiding patterns after cesarean delivery without catheterization. Updated protocols that align with evolving surgical practices and patient care standards are warranted. 
